# A Phenomenological Model for Electrical Transport Characteristics of MSM Contacts Based on GNS

**DOI:** 10.3390/mi14010184

**Published:** 2023-01-11

**Authors:** Meisam Rahmani, Hassan Ghafoorifard, Mohammad Taghi Ahmadi

**Affiliations:** 1 Department of Electrical and Computer Engineering, Buein Zahra Technical University, Buein Zahra 34517-45346, Iran; 2 Department of Electrical Engineering, Amirkabir University of Technology, 424 Hafez Ave., Tehran 15914, Iran; 3 Device Modelling Group, School of Engineering, University of Warwick, Coventry CV4 7AL, UK; 4 Nanotechnology Research Center, Nano-Physic Group, Physics Department, Urmia University, Urmia 57147, Iran

**Keywords:** GNS, MSM contacts, FET, surface potential, subthreshold slope, DIBL, current-voltage characteristic

## Abstract

Graphene nanoscroll, because of attractive electronic, mechanical, thermoelectric and optoelectronics properties, is a suitable candidate for transistor and sensor applications. In this research, the electrical transport characteristics of high-performance field effect transistors based on graphene nanoscroll are studied in the framework of analytical modeling. To this end, the characterization of the proposed device is investigated by applying the analytical models of carrier concentration, quantum capacitance, surface potential, threshold voltage, subthreshold slope and drain induced barrier lowering. The analytical modeling starts with deriving carrier concentration and surface potential is modeled by adopting the model of quantum capacitance. The effects of quantum capacitance, oxide thickness, channel length, doping concentration, temperature and voltage are also taken into account in the proposed analytical models. To investigate the performance of the device, the current-voltage characteristics are also determined with respect to the carrier density and its kinetic energy. According to the obtained results, the surface potential value of front gate is higher than that of back side. It is noteworthy that channel length affects the position of minimum surface potential. The surface potential increases by increasing the drain-source voltage. The minimum potential increases as the value of quantum capacitance increases. Additionally, the minimum potential is symmetric for the symmetric structure (*V_fg_* = *V_bg_*). In addition, the threshold voltage increases by increasing the carrier concentration, temperature and oxide thickness. It is observable that the subthreshold slope gets closer to the ideal value of 60 mV/dec as the channel length increases. As oxide thickness increases the subthreshold slope also increases. For thinner gate oxide, the gate capacitance is larger while the gate has better control over the channel. The analytical results demonstrate a rational agreement with existing data in terms of trends and values.

## 1. Introduction

**Figure 1:**
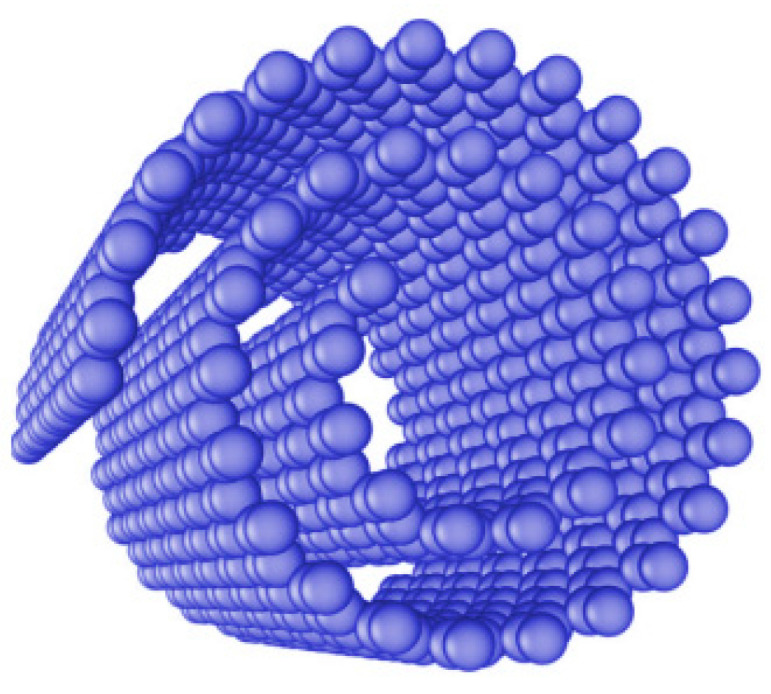
GNS which is made by rolling graphene into a spiral form.

Nowadays, the search for new nano-materials has an enormous interest owing to their significant applications in our life [1,2,3,4,5]. Nano-materials including carbon nanotube, mono and multilayers graphene and phosphorene have been predicted many years ago, for there great promise in different applications, such as electronics, energy harvesting, spintronic devices, molecular sensors, gene and drug distribution systems, lasers, ion channels, batteries, solar cells, photocatalysis, polymer composites, and high-frequency nanoelectromechanical resonators [6,7,8,9,10,11,12,13,14,15,16]. These materials reveal startling physical, mechanical, thermal, electrical, and chemical properties such as high surface area, strong mechanical strength, good thermal conductivity, excellent electrical conductivity, high charge carrier mobility, good optical transparency and ease of biological as well as chemical functionalization that leads to great opportunities for implementing into a broad area of transistor and sensor applications [17,18,19,20,21,22,23,24,25,26]. Graphene nanoscroll (GNS), as a well-known stable elemental semiconducting material, has attracted strong scientific and technological interest in recent years [27,28,29,30]. GNS with unique electronic, transport, thermoelectric, optoelectronics and mechanical characteristics such as ballistic transport, stability, large surface-to-volume ratio, high conductivity, high flexibility and biocompatibility has great potential in material science, energy storage, biosensing, biocompatibility, bio-engineering and designing nanoelectronic devices. GNS as a superlative semiconductor promises potential applications in the diodes, FETs, gas and biosensors [27,28,29,30]. The influence of GNS on transistor applications has been reported and its geometry effect on FET performance has been investigated [31]. It has been concluded that the chirality number plays an important role compared to the other parameters. GNS has emerged as a new category of quasi one dimensional (1D) belonging to the carbon-based components, which is made by rolling a graphene sheet to form an open cylindrical structure. GNS with a tubular structure similar to that of the open multi-walled carbon nanotube (MWCNT), has various morphologies such as armchair (*n*, *n*), zigzag (*n*, 0), and chiral (*n*, *m*) [32]. The structure of GNS is illustrated in Figure 1.

**Figure 2:**
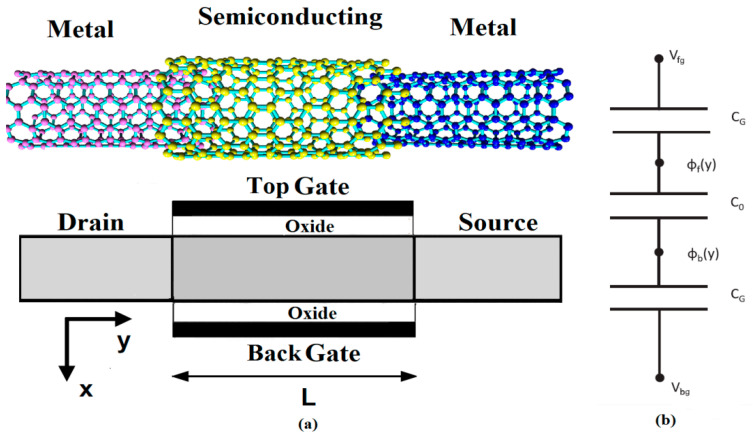
(**a**) The scheme of MSM contacts in the structure of the proposed GNS-based double gate FET, (**b**) equivalent circuit of device electrostatics.

Overcoming the chirality and diameter control issues makes the nanomaterial a proper candidate to be utilized in channel of FETs. High carrier mobility in GNS combined with the ability to modulate the carrier concentration causes high field-effect mobility in nanoscale FETs, chiefly those based on low noise and high-frequency operation. Figure 2 shows the scheme of metal-semiconducting-metal (MSM) junctions and a structure of the proposed GNS-based double gate FET with a 20 nm channel length. The width of ribbons and the shape of edges are two important factors in the band gap of GNS. The structure is proposed based on dependence of the energy band gap of armchair GNS on width of ribbon and the shape of its edges. The proposed GNS-FET structure is made from a semiconducting GNS as the channel, and metal GNS as the source and drain contacts, respectively. GNS with extremely thin body thickness allow excellent electrostatic control. Therefore, it can improve the gate control, which is desirable for the ultimately scaled transistor to reduce the short channel effects. It is noteworthy that the gate metal work function is considered so that the zero potential point in the channel stays in the middle of the band gap at an equilibrium condition. The transport properties of GNS nanoribbons can be controlled by the staggered sublattice potential induced by a perpendicular electric field. The required electric field can be produced by applying different voltages to the gate. If the electric field applied is not perpendicular to the GNS layer, the staggered potential is reduced, and a larger electric field is required to produce the same effect on the conductance.

In the previously reported works, the electrical transport characteristics of nanoscale FETs have been investigated [33,34,35,36,37,38]. Analytical models for threshold voltage and subthreshold behavior of double gate bilayer graphene FET have been explored [33]. The current developments and future prospects for 2D materials-based nanoscale tunneling FETs have been studied [34]. Tunable electronic transport characteristics of surface-architecture-controlled ZnO nanowire FETs have been reviewed [35]. The semi-analytical models of momentum relaxation mean free time and path and ionization coefficient of trilayer graphene nanoribbon-based FETs have been investigated [36]. Threshold voltage manipulation of ZnO-graphene oxide hybrid thin film transistors via Au nanoparticles doping has been studied [37]. In addition, controlling the threshold voltage of a semiconductor FET by gating its graphene gate has been investigated [38]. This paper is organized as follows: in Section 2, the analytical method and proposed models are presented. The obtained analytical results and main findings are shown in Section 3, and concluding remarks are given in Section 4.

## 2. Analytical Modeling

### 2.1. Analytical Models for Surface Potential and Subthreshold Slope

In order to model the characteristics of GNS such as carrier concentration and quantum capacitance, its *E-k* relationship is adopted from [32]. The carrier concentration is analytically modeled as Equation (1), which is related to the Fermi-Dirac integral.

(1) $$n=\int_{E_{C}}^{E_{top}}\frac{1}{1+e^{\frac{E-E_{F}}{K_{B}T}}}\left(\frac{1}{3ta_{cc}{}^{2}\pi }\right.\left(\frac{4E}{3ta_{cc}{}^{2}}\right.\pm {\left.\left.\frac{20}{3a_{cc}{}^{2}}\right)\right)}^{-\frac{1}{2}}dE$$

where *a_cc_* is the distance between adjacent carbon atoms, *t* is the hopping energy and *T* is the temperature. Using the common Poisson’s equation the potential distribution, ϕ(x,y), for any point (*x*,*y*) of channel is given by [33,39,40,41]

(2) $$\begin{array}{l} \frac{\partial^{2}\phi (x,y)}{\partial x^{2}}+\frac{\partial^{2}\phi (x,y)}{\partial y^{2}}=\frac{q(N_{D}+n_{i})}{\varepsilon_{g}}\\ 0\leq x\leq t_{ch},0\leq y\leq L \end{array}$$

where $\varepsilon_{g}$ is the dielectric constant of GNS; *q* is the electron charge; *N_D_* [in cm^−3^] is the doping concentration and $n_{i}=[n/t_{ch}]$ is the intrinsic carrier concentration where *n* is the 2D carrier concentration of GNS, so

(3) $$\frac{\partial^{2}\phi (x,y)}{\partial x^{2}}+\frac{\partial^{2}\phi (x,y)}{\partial y^{2}}=\frac{q(N_{D}+\int_{E_{C}}^{E_{top}}\frac{1}{t_{ch}(1+e^{\frac{E-E_{F}}{K_{B}T}})}\left(\frac{1}{3ta_{cc}{}^{2}\pi }\right.\left(\frac{4E}{3ta_{cc}{}^{2}}\right.\pm {\left.\left.\frac{20}{3a_{cc}{}^{2}}\right)\right)}^{-\frac{1}{2}}dE)}{\varepsilon_{g}}$$

The 2D potential distribution along the vertical direction of the channel for the subthreshold region is commonly approximated by a simple parabolic function of

(4) $$\phi (x,y)=P_{0}(y)+P_{1}(y)x+P_{2}(y)x^{2}$$

where coefficients *P*_0_, *P*_1_ and *P*_2_ are only the functions of *y*. To have better insight into the proposed device operation, the electrostatic of the device is illustrated in Figure 2. Therefore, the boundary conditions for Equation (2) are defined based on the 1D Gauss’s law and the continuity of the electrostatic potential as

$$\varepsilon_{g}\frac{d\phi (x,y)}{dx}\left|{}_{x=0}\right.=(\phi_{f}(y)-V_{fg}^{\prime })C_{G}+(\phi_{b}(y)-\phi_{f}(y))C_{0}$$

(5) $$\varepsilon_{g}\frac{d\phi (x,y)}{dx}\left|{}_{x=t_{ch}}\right.=-(\phi_{b}(y)-V_{bg}^{\prime })C_{G}-(\phi_{b}(y)-\phi_{f}(y))C_{0}$$

where $\phi_{f}(y)=\phi (0,y)$, $\phi_{b}(y)=\phi (t_{ch},y)$ are the potential functions along the front and back oxide-channel interfaces, respectively. Additionally, $V_{fg}^{\prime }=V_{fg}-V_{fb},V_{bg}^{\prime }=V_{bg}-V_{fb}$ is the potential on front (back) channel surface and $V_{fb}=\phi_{m}-[\frac{\chi_{g}}{q}+\frac{E_{g}}{q}+\frac{K_{B}T}{q}\ln(\frac{N_{D}}{n_{i}})]$ is the flat band voltage, in which $\phi_{m}$ is the metal work function, $\chi_{g}$ is the electron affinity, *K_B_* is the Boltzmann constant and *T* is the temperature. The coefficients *P_i_* (*i* = 0, 1, 2) can be determined by applying the boundary conditions in Equation (4) as

(6) $$P_{0}(y)=\phi_{f}(y)$$

(7) $$P_{1}(y)=\frac{C_{G}}{\varepsilon_{g}}(\phi_{f}(y)-V_{fg}^{\prime })+\frac{C_{0}}{\varepsilon_{g}}(\phi_{b}(y)-\phi_{f}(y))$$

(8) $$P_{2}(y)=C_{G}(V_{fg}^{\prime }-\phi_{f}(y))+\frac{-D[C_{G}(\phi_{f}(y)-V_{fg}^{\prime })-K]}{A}$$

where *A*, *K* and *D* are presented in Appendix A. In addition, $C_{ch}=\varepsilon_{g}/t_{ch}$ is the channel capacitance and $C_{G}=(C_{ox}C_{q})/(C_{ox}+C_{q})$ is the capacitance seen by the gate, where $C_{ox}=\varepsilon_{ox}/t_{ox}$ ($\varepsilon_{ox}$ is the oxide dielectric) and *C_q_* is the quantum capacitance. The quantum capacitance describes the effect of the conduction and the valance bands movement on the channel charge. To model the quantum capacitance, the relation ($C_{q}=e^{2}\frac{\partial n}{\partial E}$) is used. So, the quantum capacitance is analytically modeled as

(9) $$C_{q}=e^{2}\frac{\partial }{\partial E}[\int_{E_{C}}^{E_{top}}\frac{1}{1+e^{\frac{E-E_{F}}{K_{B}T}}}\left(\frac{1}{3ta_{cc}{}^{2}\pi }\right.\left(\frac{4E}{3ta_{cc}{}^{2}}\right.\pm {\left.\left.\frac{20}{3a_{cc}{}^{2}}\right)\right)}^{-\frac{1}{2}}dE]$$

By solving the differential equation for $\phi_{f}(y)$, ($\frac{\partial^{2}\phi_{f}(y)}{\partial^{2}y}-\alpha_{1}\phi_{f}(y)=\beta_{1}$), and by setting $\phi_{x}(y)=\phi (x^{\prime },y)$ as the potential at depth $x^{\prime }$ of the channel, the top gate surface potential $\phi_{f}(y)$ can be obtained as

(10) $$\phi_{f}(y)=\frac{1}{1+\frac{C_{G}}{\varepsilon_{g}}x^{\prime }-\frac{C_{G}(1+D)}{A}x^{\prime }{}^{2}}\times \left(\phi_{x}(y)+\frac{K-C_{G}V_{fg}^{\prime }}{\varepsilon_{g}}x^{\prime }-\frac{C_{G}(V_{fg}^{\prime }-V_{bg}^{\prime })+KD}{A}x^{\prime }{}^{2}\right)$$

where

$$C_{G}=\frac{\frac{e^{2}\varepsilon_{ox}}{t_{ox}}\left(\frac{\partial }{\partial E}[\int_{E_{C}}^{E_{top}}\frac{1}{1+e^{\frac{E-E_{F}}{K_{B}T}}}\left(\frac{1}{3ta_{cc}{}^{2}\pi }\right.\left(\frac{4E}{3ta_{cc}{}^{2}}\right.\pm {\left.\left.\frac{20}{3a_{cc}{}^{2}}\right)\right)}^{-\frac{1}{2}}dE]\right)}{\frac{\varepsilon_{ox}}{t_{ox}}+\left(e^{2}\frac{\partial }{\partial E}[\int_{E_{C}}^{E_{top}}\frac{1}{1+e^{\frac{E-E_{F}}{K_{B}T}}}\left(\frac{1}{3ta_{cc}{}^{2}\pi }\right.\left(\frac{4E}{3ta_{cc}{}^{2}}\right.\pm {\left.\left.\frac{20}{3a_{cc}{}^{2}}\right)\right)}^{-\frac{1}{2}}dE]\right)}$$

and $\alpha_{1}$ and $\beta_{1}$ are presented in Appendix A. The back gate surface potential $\phi_{b}(y)$ can be also given by finding its correlation with front gate surface potential, where the potential distribution bellow the subthreshold region is assumed as a straight line ($\phi_{b}(y)=\phi_{f}(y)-\frac{C_{0}(V_{fg}^{\prime }-V_{bg}^{\prime })}{C_{G}-C_{0}}$). In addition, the subthreshold slope can be defined as

(11) $$SS=\frac{KT}{q}\ln10{\left(\frac{d\phi_{\min,x}}{dV_{fg}}\right)}^{-1}$$

where $\phi_{\min,x}$ is the minimum potential at depth *x* of the channel and is determined based on the position of the virtual cathode along the channel.

### 2.2. Analytical Model for Electrical Transport Characteristics and DIBL

In the MSM contacts, electrons can be injected directly from the metal into the empty space in the semiconductor. When electrons flow from the valence band of the semiconductor into the metal, there would be a result similar for holes injected into the semiconductor. So, the establishment of an excess minority carrier hole in the vicinity is observed [42,43,44,45]. The current moves mainly from the drain to the source which consists of both drift current and diffusion current. Considering the weak inversion region, the current is mainly diffusion dominated and relative to the electron absorption at the virtual cathode. The tunneling current is the main component of the whole current which requires the use of quantum transport [42,43,44,45]. The effect of the charge close to the source for an FET is most severe because it has a significant effect on the MSM and the tunneling possibility. When the charge impurity is situated at the center of the channel of an FET, the electrons are trapped by the positive charge and the source-drain current is decreased. If the charge is placed close to the drain, the electrons are collected near the drain. In this situation, low charge density near the source decreases the potential barrier at the beginning of the channel which opens up the energy gap more for flow of electrons from the source to the channel [42,43,44,45]. Electrons’ moving from the metal into the semiconductor can be defined by electron current density $J_{m\to s}$, whereas the electron current density $J_{s\to m}$ refers to the movement of electrons from the semiconductor into the metal. What determines the direction of electrons flow depends on the subscripts of the current. In other words, the conventional current direction is opposite to the electron flow. $J_{s\to m}$ is related to the concentration of carries (*n*) with velocity in the *x*-direction to subdue the barrier.

(12) $$J_{s\to m}=e\int_{-\infty }^{+\infty }\nu_{x}dn$$

where *e* is the magnitude of the electronic charge and $\nu_{x}$ is calculated based on Kinetic energy ($\nu_{x}=\sqrt{\frac{2(E-E_{c})}{m}}$). By considering $x=(E-E_{C})/K_{B}T$ and normalized Fermi energy $\eta =(E_{C}-E_{F})/K_{B}T$, the carrier concentration model can be obtained as

(13) $$n=\left(\frac{4}{9t^{2}a_{cc}{}^{4}\pi }\right){\left(K_{B}T\right)}^{\frac{1}{2}}{ℑ}_{\frac{-1}{2}}\left(\eta \right)dx$$

where ${ℑ}_{\frac{-1}{2}}\left(\eta \right)$ is the Fermi–Dirac integral of order (−1/2). Total current density can be calculated as ($J_{total}=J_{m\to s}-\frac{dJ_{s\to m}}{dx}$). High carrier mobility reported from experiments in the GNS leads to assume completely ballistic carrier transport in this material, which means the average probability of injected electron at one end that will transmit to the other end is approximately equal to one ($J_{total}=J_{m\to s}=J_{s\to m}$). Kinetic energy as a main parameter is considered over the Fermi level and the current density-voltage response of GNS-FET device is determined with respect to the carrier density and its kinetic energy as

(14) $$J_{s\to m}=\frac{\sqrt{2}e}{\sqrt{m^{*}}}\int_{-\infty }^{+\infty }\left(\frac{4}{9t^{2}a_{cc}{}^{4}\pi }\right)\left(K_{B}T\right)x^{1/2}{ℑ}_{\frac{-1}{2}}\left(\eta \right)dx$$

The dependence of the drain current on the drain-source voltage is associated with the dependence of η on this voltage given by Equation (15).

(15) $$\eta =\int_{0}^{V_{DS}}\frac{(V_{GT}-V(y))e}{K_{B}T}dv$$

where $V_{GT}=V_{GS}-V_{T}$ and V(y) is the voltage of channel in *y* direction. By solving Equation (15), the normalized Fermi energy can be defined as $\eta =\frac{e}{K_{B}T}\left[V_{GT}V_{DS}-\frac{V_{DS}^{2}}{2}\right]$. In order to obtain an analytical relation for the contact current, an explicit analytical equation for the electric potential distribution along the GNS is presented. The channel current is analytically derived as a function of various physical and electrical parameters of the device including effective mass, channel length, temperature and applied bias voltage. The current density of a GNS-FET is modeled as

(16) $$J_{s\to m}=\sqrt{\frac{2}{m^{*}}}\left(\frac{4e^{2}}{9t^{2}a_{cc}{}^{4}\pi }\right)\int_{-\infty }^{+\infty }x^{1/2}{ℑ}_{\frac{-1}{2}}\left((V_{GS}-V_{T})V_{DS}-\frac{V_{DS}^{2}}{2}\right)dx$$

According to the relationship between a current and its density, the current–voltage characteristics of the proposed device are investigated in section of results and discussion. The source to the channel barrier prevents the carriers from moving in the longitudinal direction of the channel. The drain voltage in short channel FETs directly affects the barrier height through a phenomenon called the drain-induced barrier lowering (*DIBL*). For high drain bias voltages, the *DIBL* can be considered as the drain bias-dependent subthreshold current. Considering the diffusion current as the dominant part of the drain current, the *DIBL* is given by

(17) $$DIBL=\frac{d\log\left(\sqrt{\frac{2}{m^{*}}}(\frac{4e^{2}}{9t^{2}a_{cc}{}^{4}\pi })\int_{-\infty }^{+\infty }x^{1/2}{ℑ}_{\frac{-1}{2}}\left((V_{GS}-V_{T})V_{DS}-\frac{V_{DS}^{2}}{2}\right)dx\right)}{dV_{DS}}$$

## 3. Results and Discussion

**Figure 3:**
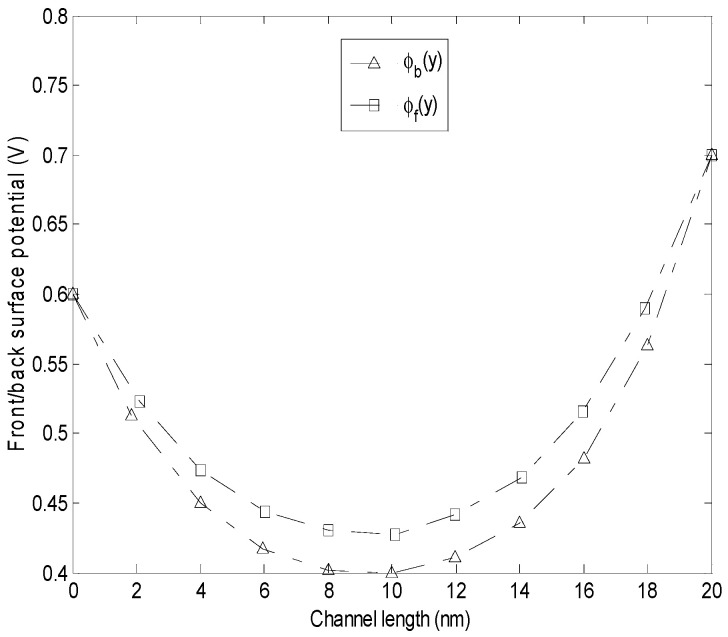
Front and back gate surface potential versus channel length.

The following parameters are used in the analytical modeling: *t_ox_ =* 1 nm, *t_ch_ =* 1.2 nm, *N_D_ =* 1 × 10^18^ cm^−3^, *n_i_ =* 5 × 10^16^ cm^−3^, *L =* 20 nm, while the flat band and gate voltages of the front and back sides are variable. The surface potential of front and back gates as a function of channel length is shown in Figure 3. Apparently, the surface potential value of front gate is higher than that of back side. It is noteworthy that channel length affects the position of minimum surface potential.

**Figure 4:**
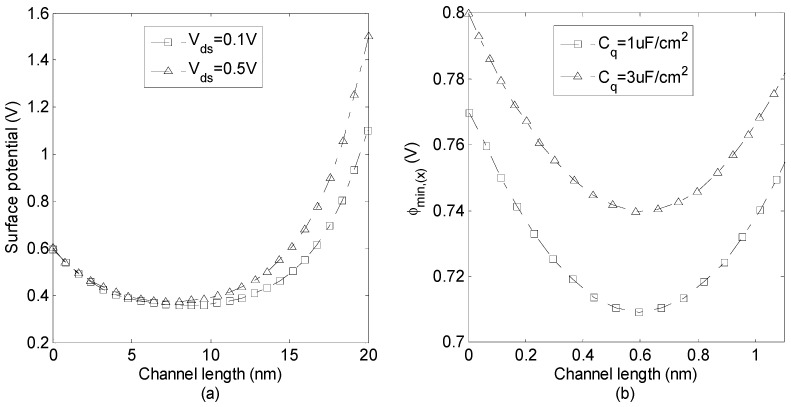
(**a**) Surface potential along the channel distance for unequal flat band/gate voltages of the front and back sides (*V_fg_* = 0.1 V, *V_bg_* = 0.2 V, *V_fb-f_* = 0.1 V, *V_fb-b_* = 0.2 V), (**b**) the minimum potential along the channel for different values of quantum capacitance (*V_fg_* = *V_bg_ =* 0.4 V).

Figure 4a indicates surface potential along the channel distance for unequal flat band/gate voltages of the front and back sides. Apparently, the surface potential increases by increasing the drain-source voltage. The variation of the minimum potential along the channel for different values of quantum capacitance is plotted in Figure 4b. As shown in Figure 4b, the minimum potential increases by increasing the value of quantum capacitance. Additionally, the minimum potential is symmetric for the symmetric structure (*V_fg_* = *V_bg_*), and *y_min_* is at the middle of the channel.

**Figure 5:**
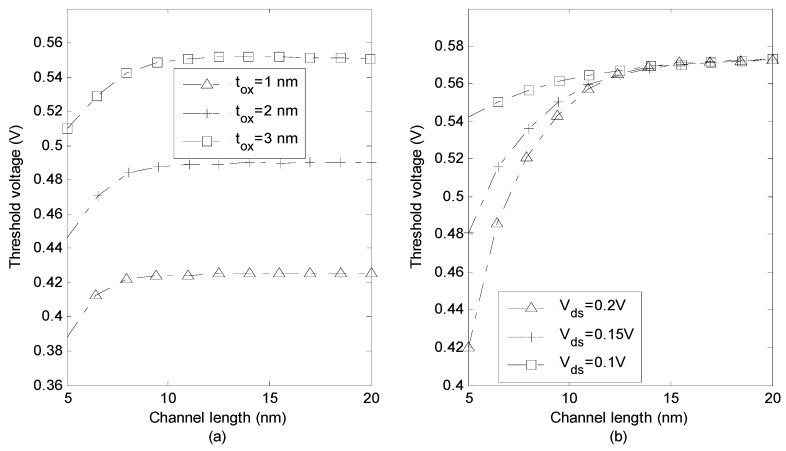
(**a**) Threshold voltage versus channel length for different values of (**a**) oxide thickness (**b**) drain-source voltage.

The effect of gate-oxide thickness on threshold voltage versus channel length is illustrated in Figure 5a. Apparently, threshold voltage increases by increased oxide thickness. This is because of the fact that gate-oxide electric field increases as oxide thickness decreases. The variation of threshold voltage along the channel for different drain–source voltages is plotted in Figure 5b. It can be seen that the threshold voltage reduces as the voltage increases. It is noteworthy that the effect of drain–source voltage is more significant for the source side of the channel.

**Figure 6:**
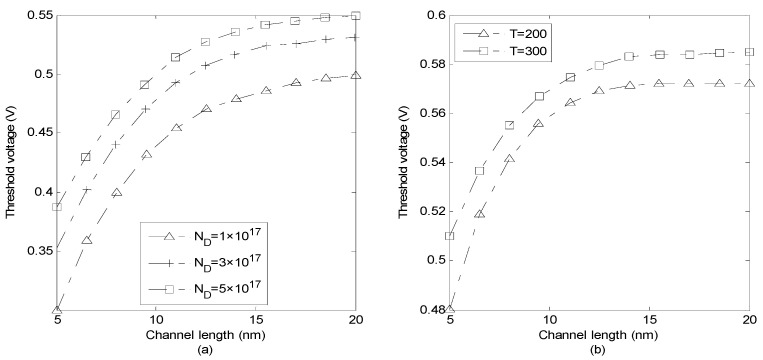
(**a**) Threshold voltage versus channel length for different (**a**) doping concentrations (**b**) temperatures.

The doping concentration effect on the threshold voltage is indicated in Figure 6a. It seems that, as the concentration increases the threshold voltage also increases. This is due to the fact that source–channel barrier increases by increasing doping concentration which results in threshold voltage increment. It is also revealed that threshold voltage increases as channel length increases because of the increment in the electric field effect on the depletion regions of source and drain junctions. Figure 6b shows the threshold voltage along the channel for two different temperatures. The profile of voltage is the same for both values of temperature, in which the threshold voltage increases as the temperature increases.

**Figure 7:**
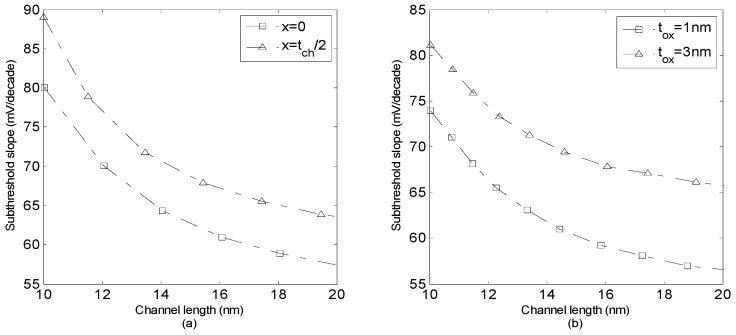
Subthreshold slope versus channel length for (**a**) the front surface and the middle of channel, (**b**) different values of oxide thickness (*V_ds_* = 0.2 V).

Figure 7a illustrates subthreshold slope versus channel length for two cases *x* = 0 and *x* = *t_ch_*/2 with *L* varying from 10 to 20 nm. The values of the subthreshold slope demonstrate a better top gate control on the front side of the channel. It is observable that the subthreshold slope gets closer to the ideal value of 60 mV/dec as the channel length increases. The obtained result of subthreshold slope resembles the better gate control on the GNS channel. However, the subthreshold slope increases for the shorter channel length devices. The effect of oxide thickness on the subthreshold slope is investigated in Figure 7b. Apparently, as oxide thickness increases the subthreshold slope also increases. For thinner gate oxide, the gate capacitance is larger while the gate has better control over the channel. Furthermore, for the thinner *t_ox_* and shorter channel devices, the subthreshold slope degradation is mainly a result of the direct source to drain tunneling. On the other hand, as the quantum capacitance is in series with *C_ox_*, the overall gate capacitance becomes smaller than *C_ox_*. Therefore, the gate control over the channel declines for larger values of *C_q_* and the subthreshold increases. It is also observed that subthreshold slope decreases as channel length increases.

**Figure 8:**
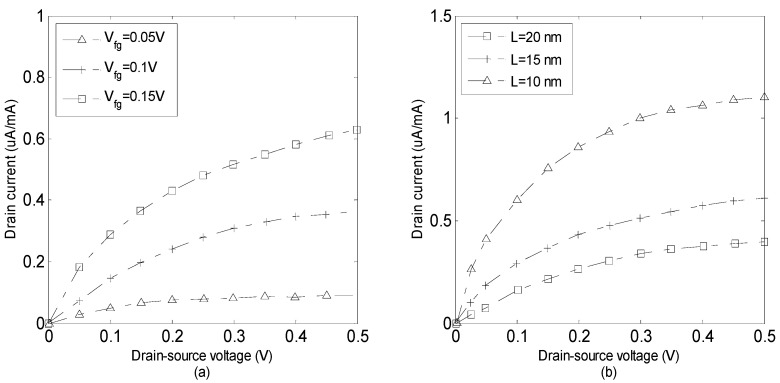
Current-voltage characteristic for different values of (**a**) front gate voltage (*V_bg_ =* 0 V, *L =* 10 nm). (**b**) channel length (*V_fg_ =* 0.2 V, *V_bg_ =* 0 V).

The current–voltage characteristics of the device are presented in Figure 8a,b. Figure 8a points out strong gate–source voltage dependence of the I–V characteristic showing that the drain current increases as the gate voltage increases. In other words, a greater value of drain current results as front gate voltage increases from 0.05 to 0.15 V. This is because of the fact that the voltage drops through the oxide close to the drain terminal reduces as the drain voltage rises. As a result, the induced inversion charge density close to the drain also decreases. The effect of the channel length scaling on the I–V characteristic is investigated in Figure 8b. Apparently, the drain current rises substantially as the channel length decreases from 20 to 10 nm. It is notable that the scaling of the channel length improves the gate electrostatic control, creating larger transconductance and smaller subthreshold swings.

**Figure 9:**
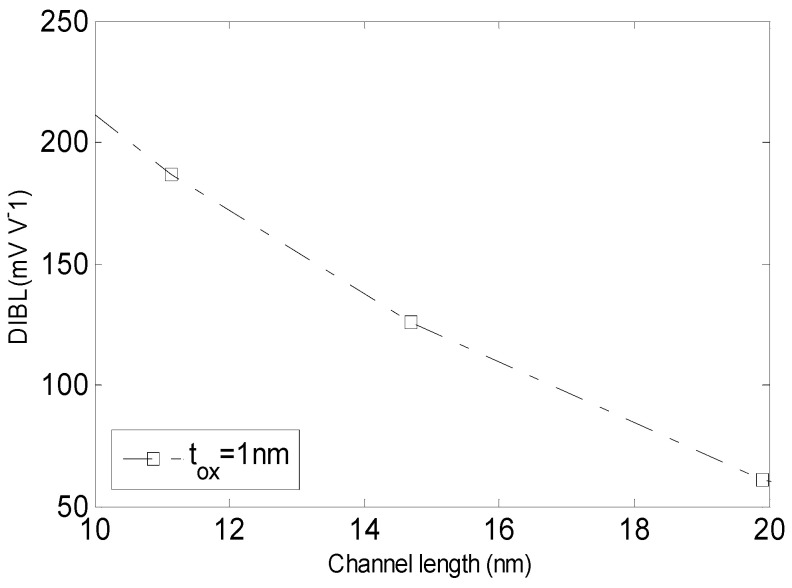
The drain induced barrier lowering dependence on the channel length.

It is noteworthy that the minimum value of surface potential and its location are dependent on the drain voltage which is a sign of *DIBL*. The variation of *DIBL* as a function of channel length is illustrated in Figure 9. It can be seen that the profile of *DIBL* decreases as channel length increases. It can be also observed that by shrinking the channel length below 20 nm, the *DIBL* effect becomes more severe. To define the physical phenomena related to the result shown in Figure 9, note that as channel length becomes shorter the depletion region increases which produces a big surface potential that decreases the barrier height and the *DIBL* effect becomes more severe.

The findings of this research demonstrate that there is a good agreement between the GNS-based device modeling and simulation results [31,32]. It can be concluded that the obtained results of the proposed analytical models and figures of merit for the proposed device showed a promising performance for transistor applications. This is because, obtained result of subthreshold slope resembles the better gate control on the GNS channel and it gets closer to the ideal value of 60 mV/dec as the channel length increases.

## 4. Conclusions

The outstanding properties of GNS are motivation for using GNS-based FETs for low-power applications. In this paper, analytical modeling of electrical transport in FET based on GNS is investigated. To this end, the characterization of the device is investigated based on analytical models of carrier concentration, quantum capacitance, surface potential, threshold voltage, subthreshold slope, DIBL and current–voltage characteristics. The effects of quantum capacitance, oxide thickness, channel length, doping concentration, temperature and voltage are also taken into account in the proposed models. According to the obtained results, the front and back gate surface potentials in the middle of channel length are about 0.4 and 0.43 V, respectively. The surface potential along the channel distance for unequal flat band/gate voltages of the front and back sides is 0.6 V (*l L*= 0). The maximum values of minimum potential for different values of quantum capacitance (1, 3 μF/cm^2^) are 0.77 and 0.8 V, respectively. The value of threshold voltage for different values of drain–source voltage is about 0.58 V in channel length of 20 nm. In this case, the value of threshold voltage reaches to maximum for different doping concentrations and temperatures. On the other hand, subthreshold slope for the front surface and the middle of channel, and different values of oxide thickness has the minimum value in the channel length of 20 nm. Additionally, the DIBL effect becomes more severe by shrinking the channel length below 20 nm. The obtained results bring new hopes for the application of GNS in high-performance transistors.

## Data Availability

The data that supports the findings of this study are available within the article and Appendix A. The results of software application are available within the article and Appendix A.

## Appendix A

$$A=2t_{ch}\varepsilon_{g}+t_{ch}^{2}(\frac{\frac{e^{2}\varepsilon_{ox}}{t_{ox}}\left(\frac{\partial }{\partial E}[\int_{E_{C}}^{E_{top}}\frac{1}{1+e^{\frac{E-E_{F}}{K_{B}T}}}\left(\frac{1}{3ta_{cc}{}^{2}\pi }\right.\left(\frac{4E}{3ta_{cc}{}^{2}}\right.\pm {\left.\left.\frac{20}{3a_{cc}{}^{2}}\right)\right)}^{-\frac{1}{2}}dE]\right)}{\frac{\varepsilon_{ox}}{t_{ox}}+\left(e^{2}\frac{\partial }{\partial E}[\int_{E_{C}}^{E_{top}}\frac{1}{1+e^{\frac{E-E_{F}}{K_{B}T}}}\left(\frac{1}{3ta_{cc}{}^{2}\pi }\right.\left(\frac{4E}{3ta_{cc}{}^{2}}\right.\pm {\left.\left.\frac{20}{3a_{cc}{}^{2}}\right)\right)}^{-\frac{1}{2}}dE]\right)}+C_{0})$$

$$K=C_{0}^{2}\left(\frac{V_{fg}^{\prime }-V_{bg}^{\prime }}{\frac{\frac{e^{2}\varepsilon_{ox}}{t_{ox}}\left(\frac{\partial }{\partial E}[\int_{E_{C}}^{E_{top}}\frac{1}{1+e^{\frac{E-E_{F}}{K_{B}T}}}\left(\frac{1}{3ta_{cc}{}^{2}\pi }\right.\left(\frac{4E}{3ta_{cc}{}^{2}}\right.\pm {\left.\left.\frac{20}{3a_{cc}{}^{2}}\right)\right)}^{-\frac{1}{2}}dE]\right)}{\frac{\varepsilon_{ox}}{t_{ox}}+\left(e^{2}\frac{\partial }{\partial E}[\int_{E_{C}}^{E_{top}}\frac{1}{1+e^{\frac{E-E_{F}}{K_{B}T}}}\left(\frac{1}{3ta_{cc}{}^{2}\pi }\right.\left(\frac{4E}{3ta_{cc}{}^{2}}\right.\pm {\left.\left.\frac{20}{3a_{cc}{}^{2}}\right)\right)}^{-\frac{1}{2}}dE]\right)}-C_{0}}\right)$$

$$D=1+t_{ch}+\frac{C_{0}t_{ch}}{\varepsilon_{g}}$$

$$\alpha_{1}=\frac{2\varepsilon_{g}^{2}\left(\frac{\frac{e^{2}\varepsilon_{ox}}{t_{ox}}\left(\frac{\partial }{\partial E}[\int_{E_{C}}^{E_{top}}\frac{1}{1+e^{\frac{E-E_{F}}{K_{B}T}}}\left(\frac{1}{3ta_{cc}{}^{2}\pi }\right.\left(\frac{4E}{3ta_{cc}{}^{2}}\right.\pm {\left.\left.\frac{20}{3a_{cc}{}^{2}}\right)\right)}^{-\frac{1}{2}}dE]\right)}{\frac{\varepsilon_{ox}}{t_{ox}}+\left(e^{2}\frac{\partial }{\partial E}[\int_{E_{C}}^{E_{top}}\frac{1}{1+e^{\frac{E-E_{F}}{K_{B}T}}}\left(\frac{1}{3ta_{cc}{}^{2}\pi }\right.\left(\frac{4E}{3ta_{cc}{}^{2}}\right.\pm {\left.\left.\frac{20}{3a_{cc}{}^{2}}\right)\right)}^{-\frac{1}{2}}dE]\right)}\right)}{\varepsilon_{g}A(\varepsilon_{g}+C_{g})x-\left(\frac{\frac{e^{2}\varepsilon_{ox}}{t_{ox}}\left(\frac{\partial }{\partial E}[\int_{E_{C}}^{E_{top}}\frac{1}{1+e^{\frac{E-E_{F}}{K_{B}T}}}\left(\frac{1}{3ta_{cc}{}^{2}\pi }\right.\left(\frac{4E}{3ta_{cc}{}^{2}}\right.\pm {\left.\left.\frac{20}{3a_{cc}{}^{2}}\right)\right)}^{-\frac{1}{2}}dE]\right)}{\frac{\varepsilon_{ox}}{t_{ox}}+\left(e^{2}\frac{\partial }{\partial E}[\int_{E_{C}}^{E_{top}}\frac{1}{1+e^{\frac{E-E_{F}}{K_{B}T}}}\left(\frac{1}{3ta_{cc}{}^{2}\pi }\right.\left(\frac{4E}{3ta_{cc}{}^{2}}\right.\pm {\left.\left.\frac{20}{3a_{cc}{}^{2}}\right)\right)}^{-\frac{1}{2}}dE]\right)}\right)(2\varepsilon_{g}+t_{ch}(\varepsilon_{g}+C_{0}))x^{2}}A(q(N_{D}+n_{i})-KD)$$

$$\beta_{1}=\frac{-((1+t_{ch})\varepsilon_{g}+C_{0}t_{ch})V_{fg}^{\prime }-\left(\frac{\frac{e^{2}\varepsilon_{ox}}{t_{ox}}\left(\frac{\partial }{\partial E}[\int_{E_{C}}^{E_{top}}\frac{1}{1+e^{\frac{E-E_{F}}{K_{B}T}}}\left(\frac{1}{3ta_{cc}{}^{2}\pi }\right.\left(\frac{4E}{3ta_{cc}{}^{2}}\right.\pm {\left.\left.\frac{20}{3a_{cc}{}^{2}}\right)\right)}^{-\frac{1}{2}}dE]\right)}{\frac{\varepsilon_{ox}}{t_{ox}}+\left(e^{2}\frac{\partial }{\partial E}[\int_{E_{C}}^{E_{top}}\frac{1}{1+e^{\frac{E-E_{F}}{K_{B}T}}}\left(\frac{1}{3ta_{cc}{}^{2}\pi }\right.\left(\frac{4E}{3ta_{cc}{}^{2}}\right.\pm {\left.\left.\frac{20}{3a_{cc}{}^{2}}\right)\right)}^{-\frac{1}{2}}dE]\right)}\right)V_{bg}^{\prime }\varepsilon_{g}}{\varepsilon_{g}A(\varepsilon_{g}+C_{g})x}-\left(\frac{\frac{e^{2}\varepsilon_{ox}}{t_{ox}}\left(\frac{\partial }{\partial E}[\int_{E_{C}}^{E_{top}}\frac{1}{1+e^{\frac{E-E_{F}}{K_{B}T}}}\left(\frac{1}{3ta_{cc}{}^{2}\pi }\right.\left(\frac{4E}{3ta_{cc}{}^{2}}\right.\pm {\left.\left.\frac{20}{3a_{cc}{}^{2}}\right)\right)}^{-\frac{1}{2}}dE]\right)}{\frac{\varepsilon_{ox}}{t_{ox}}+\left(e^{2}\frac{\partial }{\partial E}[\int_{E_{C}}^{E_{top}}\frac{1}{1+e^{\frac{E-E_{F}}{K_{B}T}}}\left(\frac{1}{3ta_{cc}{}^{2}\pi }\right.\left(\frac{4E}{3ta_{cc}{}^{2}}\right.\pm {\left.\left.\frac{20}{3a_{cc}{}^{2}}\right)\right)}^{-\frac{1}{2}}dE]\right)}\right)(2\varepsilon_{g}+t_{ch}(\varepsilon_{g}+C_{0}))x^{2}$$
